# Supplementary material for: Risk assessment of a highly pathogenic H5N1 influenza virus from mink
Source: Nat Commun. 2024 May 15;15:4112. doi: 10.1038/s41467-024-48475-y (PMC11096306; doi:10.1038/s41467-024-48475-y)
Supplement: Supplementary file 1 — Supplementary Information [file 41467_2024_48475_MOESM1_ESM.pdf]

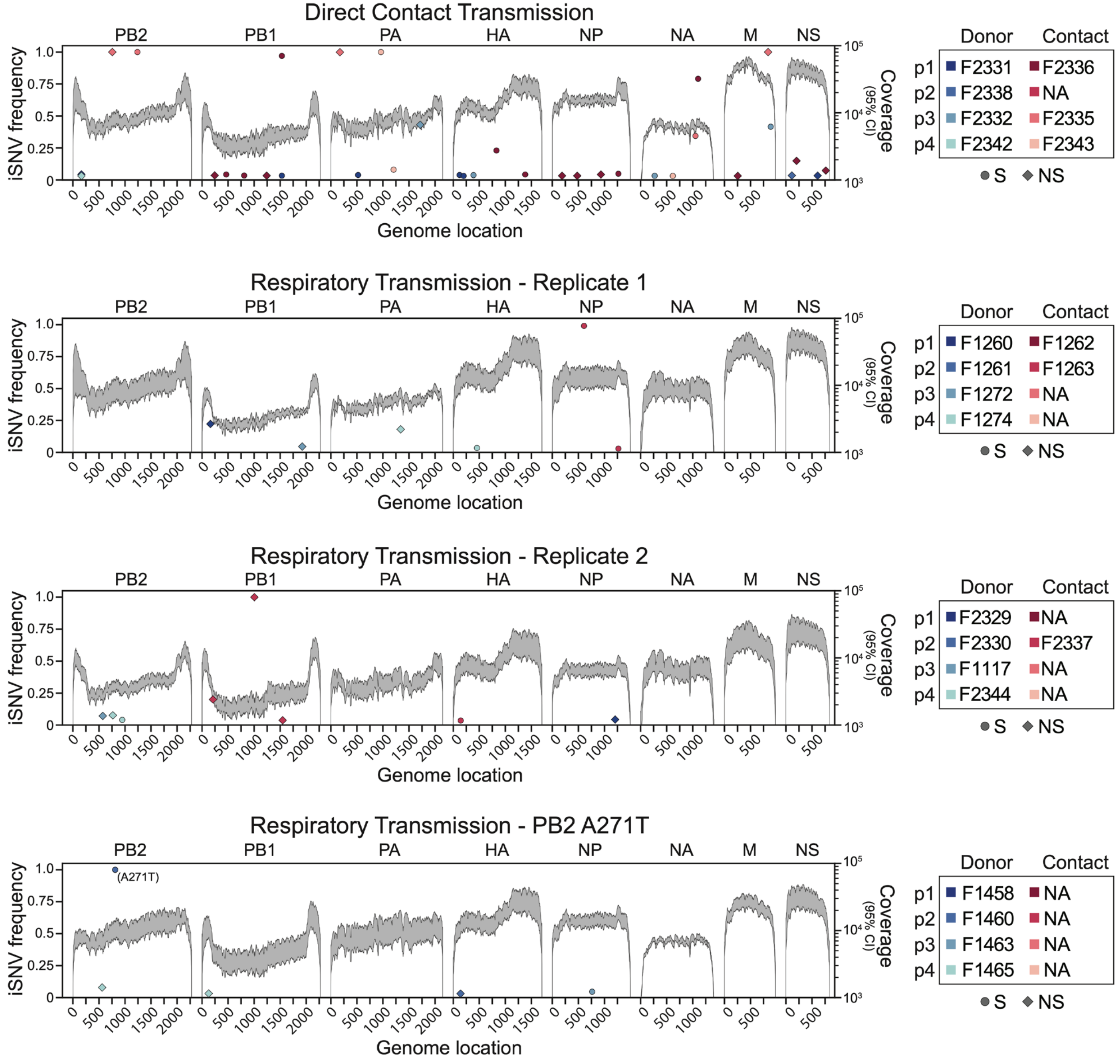

Figure S1. Intra-host Single-Nucleotide Variants (iSNV) in A/mink (H5N1) shed by infected donor and contact ferrets during transmission experiments. Shown are the iSNV identified and sequencing coverage across the full viral genome in nasal wash samples at the time of peak viral replication for individual animals. vRNA was isolated from nasal wash samples and subject to next-generation sequencing. Top panel displays iSNVs identified during contact transmission of wild-type A/mink (H5N1). Middle panels show iSNVs identified during airborne transmission study 1 and 2 with wild-type A/mink (H5N1), respectively. Bottom panel displays iSNV identified during airborne transmission of A/mink (H5N1) with PB2 A271T. Individual donor and contact ferrets are represented in each panel by different shades of blue and red, respectively. Non-synonymous mutations are denoted by diamond symbols, and synonymous mutations are denoted by circle symbols.

Table S1. Intra-host single nucleotide variants (iSNV) identified in A/mink (H5N1) viruses during transmission experiments.

Transmission Experiment	Non-Synonymous or Synonymous Change	Gene Segment	Nucleotide Change	Protein	Nucleotide Frequencing								
					Virus Inoculum	Transmission Pair 1		Transmission Pair 2		Transmission Pair 3		Transmission Pair 4	
						Donor	Contact	Donor	Contact	Donor	Contact	Donor	Contact
Direct Contact Transmission	Non-Synonymous	PB2	A163C	PB2-Y54S (NS)	Y	0.04 (S)						0.03 (S)	
		PB2	G756A	PB2-D252N (NS)	D						1 (N)		
		PB1*	T243C	PB1-Y81H (NS)	Y		0.03 (H)						
		PB1	G1241A	PB1-M413I (NS)	M		0.03 (I)						
		PA	A175G	PA-E58G (NS), PA-X-E58G (NS)	E,E						1 (G,G)		
		PA	A1716G	PA-I572V (NS)	I					0.43 (V)			
		NP	T187C	NP-I62T (NS)	I		0.03 (T)						
		NP	A478G	NP-D159G (NS)	D		0.03 (G)						
		NP	G933A	NP-V311M (NS)	V		0.04 (M)						
		M	G255A	M1-G85R (NS)	G		0.03 (R)						
		M	G838A	M2-V50I (NS)	V						1 (I)		
		NS	G114A	NS1-D38N (NS)	D			0.03 (N)					
		NS	C204A	NS1-L68M (NS)	L		0.15 (M)						
		NS*	G609A	NS1-G203R (NS)	G	0.03 (R)							
		NS	T766G	NEP-F98V (NS)	F		0.07 (V)						
	Synonymous	PB2	A1238G	PB2-412A (S)	A						1 (A)		
		PB1**	T243C	PB1-F2-49D (S)	D		0.03 (D)						
		PB1	A464G	PB1-154L (S)	L		0.04 (L)						
		PB1	A809G	PB1-269G (S)	G		0.03 (G)						
		PB1	C1532T	PB1-510S (S)	S	0.03 (S)							
		PA	G524A	PA-174L (S), PA-X-174L (S)	L,L	0.04 (L,L)							
		PA	T962C	PA-320N (S)	N								1 (N)
		PA	G1205A	PA-401S (S)	S								0.08 (S)
		HA	T122C	HA-40T (S)	T	0.04 (T)							
		HA	T200C	HA-66I (S)	I	0.03 (I)							
		HA	T389C	HA-129I (S)	I					0.04 (I)			
		HA	C830T	HA-276D (S)	D		0.23 (D)						
		HA	G1382A	HA-460K (S)	K		0.04 (K)						
		NP	G1268A	NP-422A (S)	A		0.05 (A)						
		NA	T269C	NA-89S (S)	S					0.03 (S)			
		NA	G617A	NA-205L (S)	L								0.03 (L)
		NA	T1052C	NA-350F (S)	F						0.34 (F)		
		M	A888G	M2-66G (S)	G					0.42 (G)			
		NS**	G609A	NEP-45G (S)	G	0.03 (G)							
Respiratory Transmission - Replicate 1	Non-Synonymous	PB1	A161C	PB1-K53N (NS), PB1-F2-N22T (NS)	K,N	0.22 (N,T)							
		PB1	A1924G	PB1-N641S (NS)	N					0.05 (S)			
		PA	G1341A	PA-A447T (NS)	A							0.18 (T)	
	Synonymous	HA	A455G	HA-151P (S)	P							0.03 (P)	
		NP	C608T	NP-202D (S)	D				0.99 (D)				
		NP	G1268A	NP-422A (S)	A				0.03 (A)				
Respiratory Transmission - Replicate 2	Non-Synonymous	PB2	G573A	PB2-E191K (NS)	E					0.07 (K)			
		PB2	A766G	PB2-D255G (NS)	D							0.08 (G)	
		PB1	G210T	PB1-G70W (NS), PB1-F2-M38I (NS)	G,M				0.2 (W,I)				
		PB1	G1005A	PB1-V335I (NS)	V				1 (I)				
		PB1	A1548G	PB1-I516V (NS)	I				0.04 (V)				
		NP	G1206A	NP-A402T (NS)	A	0.04 (T)							

	Synonymous	PB2	T947C	PB2-315G (S)	G							0.04 (G)	
		HA	C144T	HA-48L (S)	L				0.04 (L)				
Respiratory Transmission - PB2 A271T	Non-Synonymous	PB2	G561A	PB2-E187K (NS)	E							0.08 (K)	
		PB1	T127C	PB1-V42A (NS), PB1-F2-S11P (NS)	V,S							0.03 (A,P)	
		HA	A141T	HA-I47L (NS)	I			0.03 (L)					
	Synonymous	NP	C764T	NP-254D (S)	D					0.05 (D)			

Notes
Viral RNA was isolated from the nasal wash of each animal at the time of peak viral shedding.
Grey shading denotes no transmission to contact
Red font indicates mutations non-synonymous mutations present at a frequency greater than or equal to 5%
* denotes mutation also confers a synonymous change in another gene expressed from this segment.
** denotes mutation also confers a non-synonymous change in another gene expressed from this segment.

Table S2. Maximum change in ferret body temperature during dose de-escalation study.

Group	Ferret Number	Baseline Temp. (°C)	Maximum Temp. (ºC)	Change in Temp. (ºC) from Baseline	Day (p.i.) of Maximum Temp.
10^0	1265*	37.9	38.9	1.0	1
	382	37.8	40.3	2.5	6
	1279	39.0	41.1	2.1	3
	1283	37.2	40.1	2.9	7
10^1	1267	37.6	39.7	2.1	4
	1268	38.7	41	2.3	5
	1282	39.0	40.7	1.7	6
	1285	38.6	40.8	2.2	5
10^2	1264	38.7	40.7	2.0	6
	1266	38.6	39.8	1.2	3
	1277	39.4	40.6	1.2	4
	1278	38.4	40.6	2.2	3
10^3	1256	38.3	40.5	2.2	7
	1258	37.2	39.5	2.3	6
	1270	38.2	41.2	3	5
	1276#	38.2	40.2	2	6

Notes: *Did not shed virus or display symptoms, #Shed virus, but did not display symptoms
The only symptom that FT 1283 in group 10^0 displayed was fever. The first two ferrets of each group were male and second two were female.

Table S3. Daily clinical scores of ferrets during dose de-escalation study

Group	Ferret Number	Day 1			Day 2			Day 3			Day 4			Day 5			Day 6			Day 7		
		Activity	Respiratory	Other	Activity	Respiratory	Other	Activity	Respiratory	Other	Activity	Respiratory	Other	Activity	Respiratory	Other	Activity	Respiratory	Other	Activity	Respiratory	Other
10^0	1265	0	0	0	0	0	0	0	0	0	0	0	0	0	0	0	0	0	0	0	0	0
	382	0	0	0	0	0	0	0	0	0	0	0	0	0	0	0	0	0	0	0	1	2
	1279	0	0	0	0	0	0	0	0	0	1	0	0	1	0	0	1	0	0	1	0	1
	1283	0	0	0	0	0	0	0	0	0	0	0	0	0	0	0	0	0	0	0	0	0
10^1	1267	0	0	0	0	0	0	0	0	0	1	0	0	1	0	0	1	0	0	1	1	2
	1268	0	0	0	0	0	0	0	0	0	0	0	0	1	0	0	0	0	0	0	0	0
	1282	0	0	0	0	0	0	0	0	0	0	0	0	0	0	0	0	0	0	0	0	0
	1285	0	0	0	0	0	0	0	0	0	0	0	0	0	0	0	0	0	1	0	0	1
10^2	1264	0	0	0	0	0	0	0	0	0	0	0	0	0	0	0	0	0	0	3	3	3
	1266	0	0	0	0	0	0	0	0	0	0	0	0	0	0	0	0	0	0	0	0	2
	1277	0	0	0	0	0	0	0	0	0	0	0	0	0	0	1	0	0	2	0	0	2
	1278	0	0	0	0	0	0	0	0	0	0	0	0	0	0	1	0	0	0	0	0	0
10^3	1256	0	0	0	0	0	0	0	0	0	0	0	0	0	0	0	1	0	0	2	0	0
	1258	0	0	0	0	0	0	0	0	0	0	0	0	0	0	2	1	1	2	1	1	2
	1270	0	0	0	0	0	0	0	0	0	0	0	0	0	0	0	1	0	0	1	0	0
	1276	0	0	0	0	0	0	0	0	0	0	0	0	0	0	0	0	0	0	0	0	0

Group	Ferret Number	Day 8			Day 9			Day 10			Day 11			Day 12			Day 13			Day 14		
		Activity	Respiratory	Other	Activity	Respiratory	Other	Activity	Respiratory	Other	Activity	Respiratory	Other	Activity	Respiratory	Other	Activity	Respiratory	Other	Activity	Respiratory	Other
10^0	1265	0	0	0	0	0	0	0	0	0	0	0	0	0	0	0	0	0	0	0	0	0
	382	0	1	2	1	0	2	1	0	2	3	1	0									
	1279	1	0	0	3	0	3															
	1283	0	0	0	0	0	0	0	0	0	0	0	0	0	0	0	0	0	0	0	0	0
10^1	1267	2	1	2	3	3	3															
	1268	2	0	3																		
	1282	0	0	0	3	3	3															
	1285	2	0	0																		
10^2	1264																					
	1266	0	0	2	3	3	3															
	1277	0	0	2	3	0	3															
	1278	0	0	2	0	0	2															
10^3	1256	2	1	0	2	3	2															
	1258	2	1	2	2	2	3															
	1270	2	0	0	2	2	3															
	1276	0	0	0	0	0	0	0	0	0	0	0	0	0	0	0	0	0	0	0	0	0

Notes: Grey box indicates no clincal score as animal had been euthanized. As described in the "Methods" section, 0 denotes no clinical score and 3 denotes most severe clinical score.

Table S4. Amino acid differences between select H5N1 strains isolated from mink.

Virus Strain	Viral Protein										Collection Date (year-month-day)
	PB2		PB1	PA		NA	NS1			NS2	
	138	598	480	349	716	395	73	155	C-terminus	74	
A/mink/Spain/3691-8_22VIR10586-10/2022 (H5N1)	R	T	K	E	K	A	S	A	-------	E	2022-10-18
A/mink/Spain/22VIR12774-13_3869-2/2022 (H5N1)	R	T	X*	E	N	A	P	A	WRNEMVD	G	2022-10-26
A/mink/Spain/22VIR12774-14_3869-2/2022 (H5N1)	Q	I	K	X*	K	T	S	V	-------	E	2022-10-26

Notes: Italic denotes virus strain used in this manuscript. Sequence alignment of proteins was performed using the 6 sequences published in GISAID for H5N1 viruses recovered during the mink outbreak. Sequence alignment and comparison of amino acid changes were performed using GISAID analyses tools. This table is modified from Maemura et al., 2023[26]. X* published sequence is ambiguous; however, alignment of the 6 reported virus sequences indicates the consensus amino acids are PB1 480K and PA 349E. Underline indicates amino acids that differ from A/mink/Spain/3691-8_22VIR10586-10/2022 (H5N1).
