# Supplementary material for: Risk assessment of a highly pathogenic H5N1 influenza virus from mink
Source: Nat Commun. 2024 May 15;15:4112. doi: 10.1038/s41467-024-48475-y (PMC11096306; doi:10.1038/s41467-024-48475-y)
Supplement: Supplementary file 3 — Reporting Summary [file 41467_2024_48475_MOESM3_ESM.pdf]

nature portfolio

Corresponding author(s): Troy C. Sutton

Last updated by author(s): Apr 15, 2024

Reporting Summary

Nature Portfolio wishes to improve the reproducibility of the work that we publish. This form provides structure for consistency and transparency in reporting. For further information on Nature Portfolio policies, see our Editorial Policies and the Editorial Policy Checklist.

Statistics

For all statistical analyses, confirm that the following items are present in the figure legend, table legend, main text, or Methods section.

n/a	Confirmed	
☐	☒	The exact sample size (*n*) for each experimental group/condition, given as a discrete number and unit of measurement
☐	☒	A statement on whether measurements were taken from distinct samples or whether the same sample was measured repeatedly
☐	☒	The statistical test(s) used AND whether they are one- or two-sided<br>*Only common tests should be described solely by name; describe more complex techniques in the Methods section.*
☒	☐	A description of all covariates tested
☐	☒	A description of any assumptions or corrections, such as tests of normality and adjustment for multiple comparisons
☐	☒	A full description of the statistical parameters including central tendency (e.g. means) or other basic estimates (e.g. regression coefficient) AND variation (e.g. standard deviation) or associated estimates of uncertainty (e.g. confidence intervals)
☐	☒	For null hypothesis testing, the test statistic (e.g. *F*, *t*, *r*) with confidence intervals, effect sizes, degrees of freedom and *P* value noted<br>*Give P values as exact values whenever suitable.*
☒	☐	For Bayesian analysis, information on the choice of priors and Markov chain Monte Carlo settings
☒	☐	For hierarchical and complex designs, identification of the appropriate level for tests and full reporting of outcomes
☒	☐	Estimates of effect sizes (e.g. Cohen's *d*, Pearson's *r*), indicating how they were calculated

Our web collection on statistics for biologists contains articles on many of the points above.

Software and code

Policy information about availability of computer code

Data collection	No software was used for data collection.
Data analysis	FluSAP pipeline developed in the Lowen laboratory is available at https://github.com/Lowen-Lab/FluSAP.

For manuscripts utilizing custom algorithms or software that are central to the research but not yet described in published literature, software must be made available to editors and reviewers. We strongly encourage code deposition in a community repository (e.g. GitHub). See the Nature Portfolio guidelines for submitting code & software for further information.

Data

Policy information about availability of data

All manuscripts must include a data availability statement. This statement should provide the following information, where applicable:

- Accession codes, unique identifiers, or web links for publicly available datasets
- A description of any restrictions on data availability
- For clinical datasets or third party data, please ensure that the statement adheres to our policy

All data supporting the findings of this study are contained within the manuscript, in the Supplementary files, and/or source data file. All raw sequencing data is available in NCBI's Sequence Read Archive under BioProject accession number PRJNA1098700. See www.ncbi.nlm.nih.gov/bioproject/PRJNA1098700.

Research involving human participants, their data, or biological material

Policy information about studies with human participants or human data. See also policy information about sex, gender (identity/presentation), and sexual orientation and race, ethnicity and racism.

Reporting on sex and gender	n/a
Reporting on race, ethnicity, or other socially relevant groupings	n/a
Population characteristics	n/a
Recruitment	n/a
Ethics oversight	n/a

Note that full information on the approval of the study protocol must also be provided in the manuscript.

Field-specific reporting

Please select the one below that is the best fit for your research. If you are not sure, read the appropriate sections before making your selection.

[x] Life sciences [ ] Behavioural & social sciences [ ] Ecological, evolutionary & environmental sciences

For a reference copy of the document with all sections, see nature.com/documents/nr-reporting-summary-flat.pdf

Life sciences study design

All studies must disclose on these points even when the disclosure is negative.

Sample size	No sample size calculations were performed. Four pairs of ferrets were used for transmission studies as this is the convention in the field. For infectious dose 50 studies, groups of 4 ferrets were used as this is the minimum number of ferrets that can be used to determine the infectious dose. The number of animals used in these studies is also limited by animal housing capacity inside the BSL3 facility. The number of experimental animals used is these studies and experimental replicates is consistent with established practices in the field for testing of transmission and virulence in ferrets. See Belser et al., mBio, 13(4), 2022 and Buhnerkempe et al., eLife, Sep 2;4:e07969, 2015. For mini-genome assays, sample size was chosen based on prior experimental studies using this assay and the convention in the field (Pena et al., JVirol, May;87(9):5118, 2013, and Kandeil et al., Nat Commun. 14(1):3082, 2023).
Data exclusions	No data were excluded
Replication	All attempts are replication of experiments were successful. Multiple independent experiments and replicates are detailed in the methods and figure legends. Assays were performed according to standard operating procedures and protocols to ensure uniformity among all investigators. Transmission studies were performed with 4 pairs of ferrets, and infectious dose 50% studies were performed over multiple doses with groups of 4 ferrets. For the airborne transmission study with the wild-type virus, this study was repeated a second time. Three independent mini-genome assays were performed in triplicate.
Randomization	Ferrets were divided into groups of 4 animals and each group of 4 contained 2 female and 2 male ferrets. Thus, the groups could not be generated randomly.
Blinding	Experiments could not be blinded. For transmission studies, the cages are designed with directional airflow from the infected animal to the contact. Experimenters must know which animals are the contact and donors to prevent cross-contamination. For infectious dose 50 studies, these studies could not be blinded as experimenters must be aware of the different experimental groups to prevent any cross-contamination. For mini-genome and serological assays, these assays required the use of a select agent virus or plasmids that expressed components of a select agent. To ensure compliance with regulations and biosafety protocols, the identity of the virus and cells transfected with plasmids must be known at all times.

Reporting for specific materials, systems and methods

We require information from authors about some types of materials, experimental systems and methods used in many studies. Here, indicate whether each material, system or method listed is relevant to your study. If you are not sure if a list item applies to your research, read the appropriate section before selecting a response.

Materials & experimental systems

n/a	Involved in the study	
☒	☐	Antibodies
☐	☒	Eukaryotic cell lines
☒	☐	Palaeontology and archaeology
☐	☒	Animals and other organisms
☒	☐	Clinical data
☒	☐	Dual use research of concern
☒	☐	Plants

Methods

n/a	Involved in the study	
☒	☐	ChIP-seq
☒	☐	Flow cytometry
☒	☐	MRI-based neuroimaging

Eukaryotic cell lines

Policy information about cell lines and Sex and Gender in Research

Cell line source(s)	MDCK -London line was obtained from the International Reagent Resource. 293T cells were obtained from the American Type Culture Collection.
Authentication	None of the cell lines were authenticated
Mycoplasma contamination	Cells lines are tested every other month for mycoplasma and are negative for mycoplasma.
Commonly misidentified lines (See ICLAC register)	No commonly misidentified cells lines were used.

Animals and other research organisms

Policy information about studies involving animals; ARRIVE guidelines recommended for reporting animal research, and Sex and Gender in Research

Laboratory animals	Ferrets (Mustela putorius furo), 23 weeks of age
Wild animals	No wild animals were used.
Reporting on sex	Equal numbers of male and female ferrets are used. Results are not segregated by sex due to relatively small group size.
Field-collected samples	No field samples were collected.
Ethics oversight	The Pennsylvania State University Institutional Biosafety Committee approved all research with biohazards under protocol No. 48971. Pennsylvania State University Institutional Animal Care and Use Committee (IACUC) approved all animal research protocol. The protocol number is 201800250. This information is also listed in the manuscript.

Note that full information on the approval of the study protocol must also be provided in the manuscript.
